# Supplementary material for: Nutritional supplementation, tooth crown size, and trait expression in individuals from Tezonteopan, Mexico
Source: PLoS One. 2024 Jun 6;19(6):e0305123. doi: 10.1371/journal.pone.0305123 (PMC11156277; doi:10.1371/journal.pone.0305123)
Supplement: S1 Table — Note: All teeth are uppers. M1 = first molar, M2 = second molar, I1 = first incisor, I2 = second incisor, C = canine. 1ICD = intercusp distance. See Fig 2 for specific distances. (DOCX) [file pone.0305123.s001.docx]

**S1 Table. Results from Shapiro-Wilks tests for normality.**

|  | | All Data | |
| --- | --- | --- | --- |
|  |  | W | *p*-value |
| Crown Size | M1 | 0.855 | <0.000 |
|  | M2 | 0.733 | <0.000 |
|  | I1 | 0.928 | <0.000 |
|  | I2 | 0.557 | <0.000 |
|  | C | 0.981 | <0.000 |
| Absolute Molar Intercusp Distances  (ICD^1^) | ICD1 | 0.973 | <0.000 |
|  | ICD2 | 0.974 | <0.000 |
|  | ICD3 | 0.976 | 0.084 |
|  | ICD4 | 0.924 | <0.000 |
|  | ICD5 | 0.991 | 0.766 |
|  | ICD6 | 0.981 | 0.021 |
| Relative (Size Corrected) Molar Intercusp Distances (ICD^1^) | ICD1 | 0.974 | 0.059 |
|  | ICD2 | 0.991 | 0.818 |
|  | ICD3 | 0.967 | 0.027 |
|  | ICD4 | 0.811 | <0.000 |
|  | ICD5 | 0.983 | <0.000 |
|  | ICD6 | 0.964 | 0.016 |
| Molar Tooth Traits | Cusp 5 | 0.523 | <0.000 |
|  | Carabelli | 0.852 | <0.000 |
| Anterior tooth traits | Tuburculum Dentale | 0.956 | <0.000 |

Note: All teeth are uppers. M1=first molar, M2=second molar, I1=first incisor, I2=second incisor, C=canine.

^1^ICD = intercusp distance. See Fig 2 for specific distances.
