# Supplementary material for: Nutritional supplementation, tooth crown size, and trait expression in individuals from Tezonteopan, Mexico
Source: PLoS One. 2024 Jun 6;19(6):e0305123. doi: 10.1371/journal.pone.0305123 (PMC11156277; doi:10.1371/journal.pone.0305123)
Supplement: S2 Table — Note: Breakpoints were set following Hanihara [45] and Scott et al. [42]. (DOCX) [file pone.0305123.s002.docx]

**S2 Table. Comparison of crown traits between the supplemented and non-supplemented groups.**

|  | Sample | | | |
| --- | --- | --- | --- | --- |
|  | Supplemented | | Non- Supplemented | |
| Trait | % | n | % | n |
| Cusp 5 UM1  (+ = ASUDAS 1-5^1^) | 26.4 | 9 | 56.4 | 22 |
| Cusp 5 M1 – ASUDAS 1 | 5.1 | 2 | 17.6 | 6 |
| Cusp 5 M1 – ASUDAS 2 | 10.2 | 4 | 17.6 | 6 |
| Cusp 5 M1 – ASUDAS 3 | 15.3 | 6 | 17.6 | 6 |
| Cusp 5 M1 – ASUDAS 4 | 7.6 | 3 | 17.6 | 6 |
| Cusp 5 M1 – ASUDAS 5 | 0.0 | 0 | 2.9 | 1 |
| Carabelli UM1  (+ = ASUDAS 1-7^1^) | 69.2 | 27 | 79.4 | 27 |
| Carabelli M1 – ASUDAS 1 | 30.7 | 12 | 5.8 | 2 |
| Carabelli M1 – ASUDAS 2 | 5.1 | 2 | 17.6 | 6 |
| Carabelli M1 – ASUDAS 3 | 10.2 | 4 | 17.6 | 6 |
| Carabelli M1 – ASUDAS 4 | 15.3 | 6 | 17.6 | 6 |
| Carabelli M1 – ASUDAS 5 | 7.6 | 3 | 17.6 | 6 |
| Carabelli M1 – ASUDAS 6 | 0.0 | 0 | 2.9 | 1 |
| Carabelli M1 – ASUDAS 7 | 0.0 | 0 | 0 | 0 |
| Tuberculum Dentale UI1  (+ = ASUDAS 1-6^1^) | 79.4 | 31 | 50.0 | 17 |
| Tuberculum Dentale UI2  (+ = ASUDAS 1-6^1^) | 71.7 | 28 | 59.2 | 20 |
| Tuberculum Dentale UC  (+ = ASUDAS 1-5^1^) | 97.4 | 38 | 79.4 | 27 |

^1^Breakpoints were set following Hanihara (2008) and Scott et al (2016).
