# Supplementary material for: Nutritional supplementation, tooth crown size, and trait expression in individuals from Tezonteopan, Mexico
Source: PLoS One. 2024 Jun 6;19(6):e0305123. doi: 10.1371/journal.pone.0305123 (PMC11156277; doi:10.1371/journal.pone.0305123)
Supplement: Attachment — Submitted filename: Revisions from Reviewer Comments_EBSefczek et al.docx [file pone.0305123.s003.docx]

Claudia Trandade Mattos, Ph.D.

Academic Editor

PLOS ONE

Dear Dr. Trindade Mattos and PLOS ONE Manuscript Editors,

My colleagues and I are pleased to submit our revised manuscript entitled “Nutritional supplementation, tooth crown size, and trait expression in individuals from Tezonteopan, Mexico.” We thank you, the Academic Editor, and our reviewers for your thoughtful and insightful comments. Below please find our detailed descriptions outlining how MS revisions were made in response to their feedback. Note, reviewer one had comments within the MS. Responses to those comments are included here, after their numbered comments and in the order they appear in the MS marked up document.

REVIEWER 1 COMMENT 1: Include an interobserver and intraobserver error test for scoring dental traits. This is important because their statistical test is based on grades and other studies have shown high inter- and intra- observer error in, for example, grades 1 vs 2 and this comes up in the results

RESPONSE: We added the following statement to lines 304-308: As the first author recorded all data presented here, the One-way Interclass Correlation (ICC) statistic was calculated on a sample of 30 individuals to test intraobserver reliability overall for all traits scored in this study. Using the scale set by Koo and Li [57], results of the ICC (0.87) suggest intraobserver reliability is “good” (between 0.75 and 0.90).

REVIEWER 1 COMMENT 2: we should know if observers were ‘blinded’ to the groups before they scored/measured teeth;

RESPONSE: We added the following statement in Lines 273-274 in Materials and Methods section under Study Collection header: “Observations were undertaken by the first author, who was blinded to the groups during data recording.”

REVIEWER 1 COMMENT 3: I’d like to know why only upper teeth were included;

RESPONSE: We added the following statement in lines 268-272: “In the original study [54], focus was placed on recording LEH of upper anterior teeth. Therefore, most dental casts available from the study participants were of their maxillary dentitions. To maximize sample sizes, only upper dentitions were included in the present study: upper central incisors (I1), lateral incisors (I2), canines (C), first molars (M1) and second molars (M2) were observed.”

REVIEWER 1 COMMENT 4: I’d like to know if/how the authors dealt with tooth wear

RESPONSE: We added the following statement in lines 264-266: “All dentitions were those of subadults and young adults ranging in age from 10-20 years, so there was very little wear overall. From this sample, only teeth with less than 5% estimated wear were included in the analysis”

REVIEWER 1 COMMENT 5: I’d like to see a comparison of cusp distance measurements made using digital calipers v. from digital images (as has been standard in the literature); it would be interesting to know if Carabelli's trait presence (grade 3 and above) is different between groups.

RESPONSE: As all measurements were made with digital calipers, there is internal consistency with respect to how dental dimensions and cusp distances were measured. It is possible that there are differences between photographic and caliper measurements, but these would not affect the internal consistency of the measurements done in this study. Thus, although we agree with the reviewer that this would be an interesting question to address, we did not feel that a comparison between the two methods was necessary for this manuscript.

REVIEWER 1 COMMENT: Change writing to follow “professional” terminology (tip instead of peak, central and lateral instead of first and second, etc.)

RESPONSE: Throughout the MS, any “peak” was changed to “tip”. In relation to incisors, all “first(s)” were changed to “central” and “second(s)” were changed to “lateral”

REVIEWER 1 COMMENT: The writing needs quite a bit of work. The manuscript feels like it was written by two different people. The introduction is quite good: it is clearly written and accurate. The rest of the manuscript is not as well organized, has type-os, is not as clearly written and much less professional.

RESPONSE: We made editorial changes throughout the MS to address this issue.

REVIEWER 1 COMMENT: line 216 the phrase “a pattern of increased cusp 5 expression” is ambiguous. I do not know if it refers to the frequency of cusp 5 or the expression. And I do not know to what “increased” refers – relative to what? There several examples like this I have noted in the marked up version of the manuscript.

RESPONSE: The hypothesis section was reworked (lines 142-211). The wording of this sentence was changed to “Hypothesis 4 predicts that the non-supplemented group will exhibit a greater frequency of upper cusp 5 (Prediction 4a) and exhibit upper cusp 5 of larger size (Prediction 4b) compared to the supplemented group” (lines 169-171) for clarification. Other editorial changes were made where noted in the marked up version.

REVIEWER 1 COMMENT: line 289 describes that “six lines were measured on each tooth”. I think they mean the “distances between cusp tips on each principal cusp were measured three times” but I’m not sure.

RESPONSE: The following adjustments were made to this sentence in lines 285-286: “Here, distances between pairs of principal cusp tips were measured using digital calipers. Six distances were measured for each tooth (Figure 2).”

REVIEWER 1 COMMENT: The conclusions are mixed but the authors conclude that "developmental environments in this case mild-to-moderate malnutrition, appears to influence human tooth size..." It is, in fact, supported for anterior teeth only.

RESPONSE: The concluding statements were adjusted in lines 579-582: “The findings presented here are consistent with others showing that developmental physiological stress can alter dental phenotypes [19,28,48,49,65]. Given the broad application of dental characteristics in biodistance studies [1,2,3,4,5,6,7], it may be useful to consider the impact that developmental stress might have on these traits.”

REVIEWER 1 COMMENT: Edit following the mark-up document provided

RESPONSE: Changes were made throughout the document following the editorial suggestions.

REVIEWER 1 COMMENT WITHIN MS: Line 175-177: “Physiological stress, reflected by the presence of LEH, may result in an increased frequency and greater expression of some molar accessory cusps (Riga et al., 2014). Based on the PCM, the expectation is that when space between cusps is small relative to crown size, the likelihood of accessory cusp formation increases (Hunter et al., 2010).” Why?

RESPONSE: Section was reworked as follows (lines 158-167): “This hypothesis and its corresponding predictions are based on the findings of Riga et al. [19], that environmental stress was associated with enhanced frequency and greater expression of variable molar accessory cusps. If consistent with PCM expectations [34], greater frequency and expression of the Carabelli cusp will be accompanied by reduced relative intercusp spacing between the paracone-protocone (ICD 1), paracone-metacone (ICD 2), hypocone-protocone (ICD 4), and/or metacone-protocone (ICD 6). Reduction in the relative spacing of these cusps would indicate that there were smaller inhibitory zones surrounding them during development, leaving room for the formation of a Carabelli cusp on the crown periphery [34]. If this prediction is not supported, then it is possible that under the influence of nutritional deprivation, developmental outcomes expected under the PCM are less predictable.”

REVIEWER 1 COMMENT WITHIN MS: Line 207-209 Question mark: “Skinner and Gunz (2010) found that when the absolute spacing of mandibular principal cusps hypoconulid and entoconid was reduced, cusp 6—a centrally located accessory cusp-- was more likely to be present.”

RESPONSE: Section was reworked as follows (lines 171-178): “If consistent with PCM expectations [17], this greater frequency and expression of upper cusp 5 will be accompanied by an increase in the distance (absolute and/or relative) between the metacone and hypocone (ICD 3). Here it has been hypothesized that the greater distance between the metacone and hypocone may make it possible for a cusp 5 enamel knot to form between the inhibition zones of the two principal cusps [17]. Again, if this prediction is not realized, then it is possible that under the influence of nutritional deprivation, developmental outcomes expected under the PCM are less predictable.”

REVIEWER 1 COMMENT WITHIN MS: Line 259-261 Question mark: “LEH represents episodic stress that, for anterior teeth, does not coincide with the timing of morphogenesis in first molars, which we include in this study.”

RESPONSE: Sentence changed for clarity (line 249-254): “LEH found on anterior teeth represents episodic stress that does not coincide with the timing of morphogenesis in first molars, which we include in this study. The formation of first molar enamel knots occurs prenatally, while the mineralization of anterior teeth is largely postnatal [55]. Thus, LEH on anterior teeth do not provide direct evidence of developmental stress occurring at the time of first molar morphogenesis.”

REVIEWER 1 COMMENT WITHIN MS: Line 305-307, Appendix?: “For anterior teeth, shovel (0-7), double shovel (0-6), and tuberculum dentale (0-6) were recorded (Figure 3). For molar teeth, presence and size of metacone (0-5), hypocone (0-5), cusp 5 (0-5), and Carabelli (0-7) were included (Figure 3).”

RESPONSE: There is currently no appendix and this is a small amount of information. We prefer to retain this in the methods section.

REVIEWER 1 COMMENT WITHIN MS: Line 285-290, Figure 3 caption, #s?

RESPONSE: Added “line #” before each number in the figure caption (line 310-314).

REVIEWER 1 COMMENT WITHIN MS: Line 354-355: Star and question mark: “Teeth with four cusps were then compared to teeth with more than four cusps (Table 3).”

RESPONSE: We reworked this whole analysis. Hypothesis 6 was added to clarify these data. Wording in the results section was changed as follows: Changed for clarity (line 436-449): “Hypothesis 6: Molar Trait Expression and the Patterning Cascade Model in the Entire Sample

Hypothesis 6 predicts that central accessory traits (Carabelli) and peripheral accessory traits (upper cusp 5) may form under different conditions but are dependent on the spacing of principal molar cusps. To address whether or not the present sample followed previously outlined expectations of the Patterning Cascade Model [see 17,34], teeth with four cusps were compared to teeth with five or more cusps (i.e., teeth with Carabelli and/or cusp 5; Table 7). Teeth with five cusps exhibited smaller paracone-metacone spacing compared to teeth with four cusps (Wilcoxon test absolute distance: W=1157.5, df=1, p=0.001; Wilcoxon test relative distance: W=1092, df=1, p=0.014). The same trend occurred for the protocone-hypocone distance (Wilcoxon test absolute distance: W=1067, df=1, p=0.014; Wilcoxon test relative distance: W=1051, df=1, p=0.017). Based on these results, Prediction 6d, wherein the buccal and lingual cusps are closer together resulting in an increase in accessory cusp expression, is supported, whereas the other configurations (Predictions 6a-6c) are not.”

REVIEWER 1 COMMENT WITHIN MS: Line 399-402, comment “Nope”: “This result provides partial support for Hypothesis 2b: the non-supplemented group has smaller relative inter-cusp distances than the supplemented group, and though it does not exhibit a frequency difference in the Carabelli trait, it does exhibit elevated trait expression for ASUDAS score 2 relative to ASUDAS score 1.”

RESPONSE: Changed wording for clarification (line 369-373): “The non-supplemented group did have smaller relative intercusp distances for paracone-metacone and protocone-hypocone than the supplemented group. However, the two groups exhibited no difference in the frequency of the Carabelli trait and only a minor difference in Carabelli trait expression (elevated trait expression for ASUDAS score 2 relative to ASUDAS score 1).”

REVIEWER 1 COMMENT WITHIN MS: Line 477-479, or maybe non-supplemented weren’t that bad: “It may be that the fortified milk provided to mothers in the supplemented group was not enough of a nutritional increase to result in larger molar crown sizes compared to participants in the non-supplemented group.”

RESPONSE: Added the following statement to address comment (Lines 493-495): “Infants born to mothers in the supplemented group had greater birth weights and lost less weight just after birth compared to non-supplemented-group infants suggesting that individuals in the supplemented group were better nourished than the non-supplemented individuals [38].”

REVIEWER 1 COMMENT WITHIN MS: Line 482-484, how much? Severely?: “In the case of the pigs, young individuals were deprived of “adequate” caloric intake, which in turn affected their bone growth and overall body mass (Tonge and McCance, 1973).”

RESPONSE: Section was reworked as follows (lines 480-485): “One explanation for the lack of significant differences in molar size between the two groups is that caloric differences might not have reached levels induced experimentally in the Tonge and McCance [28] pig study. In that study, pigs were under “severe caloric restriction from early in life until they were a year old” [28:2] and weighed far less at the same age as their counterparts in the control group. Experimental animals killed after the first year weighted on average 5.5 kg, while those of the comparably aged control group weighted 180 kg [28].”

REVIEWER 1 COMMENT WITHIN MS: Line 486, Did they have the same diet except supplementation?: “Non-supplemented individuals in the present study had, in addition to a comparatively poor diet (consuming roughly 300Kcal less than the supplemented group), more frequent and longer bouts of sickness, and reduced body mass compared to individuals in the supplemented group (Chavez and Martinez, 1982)”

RESPONSE: Section was reworked as follows (lines 486-501): “By contrast, differences in the caloric intake of the supplemented vs. non-supplemented group of the present study were not as severe as those in the Tonge and McCance [28] pig study. In general, Tezonteopan residents consumed an average of 725 calories per day and 19.2 grams of protein, which is less than the daily recommended quantities of 1,250 calories 32g [38:17]. Individuals in the supplemented group were given fortified milk 2-3 times per day, which brought their dietary intake of calories and protein closer to recommended levels. Non-supplemented individuals in the present study had, in addition to a comparatively poor diet, more frequent and longer bouts of sickness, and reduced body mass compared to individuals in the supplemented group [38]. Infants born to mothers in the supplemented group had greater birth weights and lost less weight just after birth compared to non-supplemented-group infants suggesting that individuals in the supplemented group were better nourished than the non-supplemented individuals [38]. Height and weight comparisons from 18 months to two years of age showed individuals in the non-supplemented group weighed less and exhibited shorter stature than their supplemented counterparts [38]. Thus, although the two groups differed nutritionally, in body weight and in growth, the nutritional differences between them may not have been great enough to affect molar crown size.”

REVIEWER 1 COMMENT WITHIN MS: Line 490, same in pigs? “Thus, despite similarities in the symptoms of malnutrition between the studies by Tonge and McCance (1973) and Chavez and Martinez (1982)…”

RESPONSE: Sentence was deleted. Section was reworked for clarity: lines 486-501.

REVIEWER 1 COMMENT WITHIN MS: Line 493-494, Why?: “Conditions present in the study by Chavez and Martinez (1982) likely represent levels of nutrition and disruptions that are more common in “real-life” situations outside of extreme experimental conditions.”

RESPONSE: Sentence was deleted. Section was reworked for clarity: lines 486-501.

REVIEWER 1 COMMENT WITHIN MS: Line 499-501, Explain why they would be wider: “These findings concur with the study by Townsend and colleagues (2003) showing that the placement of, and space between cusps are more affected by external influences during morphogenesis than are crown dimensions”

RESPONSE: Sentence worded as follows (lines 506-509): “These findings are consistent with the study by Townsend and colleagues [18] showing that the placement and space between cusps are more affected by external influences during morphogenesis than are crown dimensions, although why this would be the case is not currently clear.”

REVIEWER 1 COMMENT WITHIN MS: Line 522-525, Developmental speed?: “Thus, one possibility is that the reduction in mesiodistal principal cups distances in the non-supplemented group is related to an alteration in the strength and/or domain of *ectodin,* or some other inhibitor of secondary enamel knot formation, which would not have affected overall crown size”

RESPONSE: We are not sure how developmental speed – or the length of time during which morphogenesis occurs—might help to explain why molar crown sizes were not affected but intercusp distances were. We would be happy to add a comment about developmental speed if we knew more about the reviewer’s perspective here. The original section with the abovementioned sentence was deleted from the manuscript.

REVIEWER 1 COMMENT WITHIN MS: Line 542-544, because…hypothesized greater cusp spacing?: “The increased presence of the central accessory cusp 5 (here found only on M1s) in the non-supplemented group is consistent with the prediction that individuals experiencing nutritional deprivation will show higher frequencies of this trait.”

RESPONSE: Changed wording for clarity (line 525-530): “The higher frequency of the upper molar cusp 5 (here found only on M1s) in the non-supplemented group is consistent with the prediction that individuals experiencing nutritional deprivation will show higher frequencies of this trait, but not with the reason for that prediction (an overall increase in principal cusp spacing relative to crown size). Based on our findings, it is possible that a decrease in mediodistal distance of the principal cusps may increase the probability of forming an upper cusp 5.”

REVIEWER 1 COMMENT WITHIN MS: Line 549, weakens?: If nutritional deprivation causes changes to molecular signaling strength…”

RESPONSE: This section was deleted from the manuscript.

REVIEWER 1 COMMENT WITHIN MS: Line 559-562, relevance? Assumed malnutrition?: “In a sample of chimpanzee teeth, Smith and Boesch (2011) found that individuals who died at younger ages also exhibited more dental anomalies (supernumerary teeth, multiple agenesis, peg-shaped teeth, and malformed dentition) compared to their adult counterparts.”

RESPONSE: This section was deleted from the manuscript.

REVIEWER 1 COMMENT WITHIN MS: Line 565-566, In a similar way to this study? “Analyzing a 20^th^ century Italian sample, Riga and colleagues (2014) report individuals with multiple LEH (“stressed” individuals) expressed more variable molar traits compared to “non-stressed” individuals; specifically, they found that the M1 Carabelli cusp and M2 hypocone of “stressed” individuals were expressed in the direction of increased variability (i.e., the presence and frequency of different trait grades) and size compared to “non-stressed” individuals.”

RESPONSE: This section was deleted from the manuscript.

REVIEWER 1 COMMENT WITHIN MS: Line 580-585, Question mark: “While cusp 5 did not conform to the predictions as outlined in the hypotheses, it is possible that the combined effects of size and placement of the principal cusps created the necessary space for the development of this central cusp. If this is the case, then the presence of cusp 5 could also be explained via the PCM, albeit through an alternate set of conditions than simply the space between the metacone and hypocone.”

RESPONSE: Section was reworked as follows (lines 557-566): “Our results show that molars with accessory cusps (Carabelli cusp, upper cusp 5, or both) have closer mesiodistal spacing between the main buccal cusps (paracone and metacone) and between the main lingual cusps (protocone and hypocone). With crown size constant, this mesiodistal compression of the main cusps can result in a greater opportunity for accessory cusps to form distally (like cusp 5) or mesially (like Carabelli cusp). Given its placement between the metacone and hypocone, upper cusp 5 expression is likely sensitive to the overall proximity of the distal main cusps (metacone and hypocone) [17], which in this sample, varies more mesiodistally than buccolingually. Carabelli cusp, being as much a mesial peripheral cusp as a lingual one, is likely sensitive to the proximity of the protocone, which in this sample varies more mesiodistally than buccolingually as well.”

REVIEWER 1 COMMENT WITHIN MS: Line 602-604, question mark: “Furthermore, the single cusp peak on an incisor is nearly as wide as the base, whereas molar cusp peaks are situated within the interior of the crown area.”

RESPONSE: Section reworked as follows (line 541-555): “The anterior and posterior teeth of this study also differed in terms of dental trait expression. An increase in trait expression for double shovel (central and lateral incisors) and fully-formed tuberculum dentale with a free apex (lateral incisors and canines) were found more often in the supplemented group. While anterior teeth exhibited greater expression of traits in the supplemented groups, the reverse was true of the posterior teeth. We expected larger anterior teeth to have a higher frequency and greater expression of variable basal features possibly as a direct result of greater mesenchymal proliferation [44,46]. Alternatively, enhanced growth may indirectly increase likelihood of forming a secondary enamel knot responsible for the variable features as the dental epithelium continues to grow further downward and outward [61]. Based on observed incisor complexity in some mammals (e.g., the three-cusped upper central incisors of plesiadapids; [see 62]), it seems likely that secondary enamel knots are involved in the morphogenesis of anterior teeth as well as molars. From a developmental standpoint, since morphogens direct the downfolding of dental epithelium [40] and tooth traits with a cusp tip are associated with a secondary enamel knot [33], it is possible that the formation of basal traits such as the tuberculum dentale could be regulated by a later forming signaling center.”

REVIEWER 1 COMMENT WITHIN MS: Line 604-606, any research on this? “Therefore, it is possible that incisor size is determined by the presence of just a primary enamel knot and less by the duration or growth rate of crown area interacting with enamel knots, as seen in molars.”

RESPONSE: This section was reworked as follows (lines 545-555): “We expected larger anterior teeth to have a higher frequency and greater expression of variable basal features possibly as a direct result of greater mesenchymal proliferation [44,46]. Alternatively, enhanced growth may indirectly increase likelihood of forming a secondary enamel knot responsible for the variable features as the dental epithelium continues to grow further downward and outward [61]. Based on observed incisor complexity in some mammals (e.g., the three-cusped upper central incisors of plesiadapids; [see 62]), it seems likely that secondary enamel knots are involved in the morphogenesis of anterior teeth as well as molars. From a developmental standpoint, since morphogens direct the downfolding of dental epithelium [40] and tooth traits with a cusp tip are associated with a secondary enamel knot [33], it is possible that the formation of basal traits such as the tuberculum dentale could be regulated by a later forming signaling center.”

REVIEWER 1 COMMENT WITHIN MS: Line 629-631, question mark: “Due to the less predictable nature of teeth affected by adverse developmental conditions, we suggest that analysis of physiological stress be considered in biodistance studies that rely on dental traits.”

RESPONSE: Both reviewers made comments on this last sentence. We deleted it and ended the paragraph with the existing previous sentence (line 581-585): “Given the broad application of dental characteristics in biodistance studies [1,2,3,4,5,6,7], it may be useful to consider the impact that developmental stress might have on these traits.”

REVIEWER 2 COMMENT 1: Sample from Mexico divided into supplemented and un-supplemented groups, but it is not clear if the latter group is actually malnourished or suffer from malnutrition.

RESPONSE: Lines 220-243 were added to clarify nutritional status and differences between the supplemented and non-supplemented groups: “Chavez and Martinez chose the town of Tezonteopan for their study because residents shared similar socioeconomic situations and traditional dietary habits (primary consumption of maize and beans, with limited amounts of fresh vegetables). Monitoring mothers’ diets and the subsequent growth of their children was the focus for two reasons: 1) the maternal environment is known to greatly impact fetal growth, and 2) women in this community relied on breastmilk as a primary food source for the first two years of a child’s life (Chavez and Martinez, 1982). Women in the supplemented group began receiving dietary supplementation consisting of milk fortified with vitamins and minerals after the first missed menstrual cycle and continuing through the duration of their pregnancies and periods of breastfeeding (Chavez and Martinez, 1982). Women in the non-supplemented group consumed the traditional diet of the community without additional nutritional aid (Chavez and Martinez, 1982). Subadult participants were placed in one of two study groups: 1) a “supplemented” group consisting of more than 40 individuals who received dietary supplementation and whose mothers were given nutritional aid during pregnancy and lactation, and 2) a “non-supplemented” group consisting of more than 40 individuals and their mothers who received no added dietary components (Chavez and Martinez, 1982).

Chavez and Martinez documented community members of Tezonteopan as mild-to-moderately malnourished, which was another of the reasons this town was chosen for their study. Dr. Chavez and Ms. Martinez (1982) found that members of both the non-supplemented and supplemented groups experienced bouts of malnutrition, likely caused by recurrent infections and a poor base diet. Comparisons of the two groups showed that subadults in the non-supplemented group had lower birth weights, shorter stature, and delayed bone maturation compared to members of the supplemented group (Chavez and Martinez, 1982). Clear distinctions in growth rate and weight gain appeared between weeks 16 and 24 after birth, with individuals in the supplemented group showing greater gains in both categories compared to members of the non-supplemented group (Chavez and Martinez, 1982).”

REVIEWER 2 COMMENT 2: I get little out of tables with a statistic and a P value. If you are going to revise this manuscript, condense and improve the tables. The tables in the supplemental materials are better than the tables in the text.

RESPONSE: Raw and average values were added to in-text tables.

REVIEWER 2 COMMENT 3: For the nonmetric dental traits, nowhere do I see trait frequencies. Your table that says 4 cusps and 5 cusps is unclear. I can only presume 5 cusps mean cusp 5 is present but that is a very non-standard way to refer to this trait. The frequencies for the grades of Carabelli's trait could also be laid out more clearly. Given the small samples sizes (barely over 30), the histogram showing grade 2 is more common in the supplemented group is not convincing.

RESPONSE: Frequencies were added to in-text tables. Hypothesis 6 was added to explain expectations and to clarify the meaning of teeth with 4 cusps and 5 cusps. The histogram was deleted since raw values were added to the tables.

REVIEWER 2 COMMENT 4: I would recommend condensing this paper. It is too long relative to the modest and sometimes inconclusive findings. Condense the tables in particular and include standard statistics for measurements and trait frequencies.

RESPONSE: Tables were changed according to reviewer suggestions. Areas of the paper were condensed and clarified.

REVIEWER 2 COMMENT 5: This is your final conclusion: Due to the less predictable nature of teeth affected by adverse developmental conditions, we suggest that analysis of physiological stress be considered in biodistance studies that rely on dental traits. – First of all, your findings are not conclusive enough to lay out such a charge. Second, how exactly would you go about assessing physiological stress in biodistance studies, especially for archaeological remains.

RESPONSE: Both reviewers made comments on this last sentence. We deleted it and ended the paragraph with the existing previous sentence (line 581-583): “Given the broad application of dental characteristics in biodistance studies [1,2,3,4,5,6,7], it may be useful to consider the impact that developmental stress might have on these traits.”

REVIEWER 2 COMMENT 6: While I admire the effort to explore this subject and test the PCM for crown size and morphology, the findings are tempered by small samples. Plus, the issue of un-supplemented vs. malnourished is not clear.

RESPONSE: Clarification on nutritional status was added, as well as the uniqueness of such a sample in humans (line 222-243 and 486-501). We have also added qualifying statements regarding the size of the sample – to the abstract, discussion, and conclusion.

Again, we thank the reviewers for their insightful comments and hope we have adequately addressed their feedback and concerns. Thank you for considering our revised manuscript.

Thank you for consideration of our manuscript.

Sincerely,

Erin C. Blankenship-Sefczek, Ph.D., Alan H. Goodman Ph.D., Mark Hubbe Ph.D., John P. Hunter Ph.D., and Debbie Guatelli-Steinberg Ph.D.
